# Socioeconomic Status, Functional Recovery, and Long-Term Mortality among Patients Surviving Acute Myocardial Infarction

**DOI:** 10.1371/journal.pone.0065130

**Published:** 2013-06-03

**Authors:** David A. Alter, Barry Franklin, Dennis T. Ko, Peter C. Austin, Douglas S. Lee, Paul I. Oh, Therese A. Stukel, Jack V. Tu

**Affiliations:** 1 The Institute for Clinical Evaluative Sciences, Toronto, Ontario, Canada; 2 The Cardiac Rehabilitation and Secondary Prevention Program, Toronto Rehabilitation Institute, Toronto, Ontario, Canada; 3 The Schulich Heart Centre and the Clinical Epidemiology Unit of Sunnybrook Health Science Centre, Toronto, Ontario, Canada; 4 The Li Ka Shing Knowledge Institute of St. Michaels’ Hospital, Toronto, Ontario, Canada; 5 Department of Medicine, University of Toronto, Toronto, Ontario, Canada; 6 Cardiac Rehabilitation and Exercise Laboratories, William Beaumont Hospital, Royal Oak, Michigan, United States of America; 7 Department of Health Policy, Management and Evaluation, University of Toronto, Toronto, Ontario, Canada; Virginia Commonwealth University, United States of America

## Abstract

**Objectives:**

To examine the relationship between socio-economic status (SES), functional recovery and long-term mortality following acute myocardial infarction (AMI).

**Background:**

The extent to which SES mortality disparities are explained by differences in functional recovery following AMI is unclear.

**Methods:**

We prospectively examined 1368 patients who survived at least one-year following an index AMI between 1999 and 2003 in Ontario, Canada. Each patient was linked to administrative data and followed over 9.6 years to track mortality. All patients underwent medical chart abstraction and telephone interviews following AMI to identify individual-level SES, clinical factors, processes of care (i.e., use of, and adherence, to evidence-based medications, physician visits, invasive cardiac procedures, referrals to cardiac rehabilitation), as well as changes in psychosocial stressors, quality of life, and self-reported functional capacity.

**Results:**

As compared with their lower SES counterparts, higher SES patients experienced greater functional recovery (1.80 ml/kg/min average increase in peak V02, P<0.001) after adjusting for all baseline clinical factors. Post-AMI functional recovery was the strongest modifiable predictor of long-term mortality (Adjusted HR for each ml/kg/min increase in functional capacity: 0.91; 95% CI: 0.87–0.94, P<0.001) irrespective of SES (P = 0.51 for interaction between SES, functional recovery, and mortality). SES-mortality associations were attenuated by 27% after adjustments for functional recovery, rendering the residual SES-mortality association no longer statistically significant (Adjusted HR: 0.84; 95% CI:0.70–1.00, P = 0.05). The effects of functional recovery on SES-mortality associations were not explained by access inequities to physician specialists or cardiac rehabilitation.

**Conclusions:**

Functional recovery may play an important role in explaining SES-mortality gradients following AMI.

## Introduction

Socioeconomic status (SES) has been shown to be an important determinant of survival after acute myocardial infarction (AMI) in countries with and without universal health care. [1] The reasons for socioeconomic-mortality disparities after AMI remain unclear. [2]–[12] Available evidence has demonstrated that SES-outcome disparities have been partially attributable to differences in baseline cardiovascular risk-factor profiles that existed prior to AMI [2], [13].

Socioeconomic differences in functional capacity have been shown to partially account for SES-mortality associations in populations with suspected coronary artery disease. [14] Moreover, available evidence from our group and others have demonstrated that access to secondary prevention services such as cardiac rehabilitation and specialty physician services after AMI are poorer among socioeconomically disadvantaged than among their socially-advantaged counterparts. [15]–[17] Accordingly, one may reasonably hypothesize that socioeconomic disparities in functional capacity recovery may exist after AMI, and that such disparities may help explain why lower SES patients experience higher long-term mortality after AMI [18], [19].

Accordingly, the objective of our study was to examine the relationship between SES, self-reported functional recovery, and long-term survival following AMI. We hypothesized that differences in access to secondary prevention service delivery may help explain SES-differences in self-reported functional recovery, and accordingly, may partially account for long-term SES-mortality associations through changes in functional capacity among AMI survivors [20].

## Methods

### Health System Context

Canada's universal health insurance system provides comprehensive coverage for most medical and hospital services without user fees at point of service. Under such provisions, patients are entitled to equitable access to medical care based on medical need, regardless of age, SES, or financial circumstances. [21] Medication costs are covered by provinces for individuals 65 years of age and older and those whose annual incomes fall at or below the poverty line. However, access to multidisciplinary secondary prevention services and related interventions are severely constrained, and have not significantly changed throughout the decade. At the time of the study, cardiac rehabilitation programs served as the only available multidisciplinary secondary prevention service program in Ontario. While some cardiac rehabilitation programs required that patients pay modest administrative fees (e.g., $25 per month) for participation, the vast majority of cardiac rehabilitation programs were funded by the Ontario government, with capacity for approximately 16,000 patients per year at the time of the study period, representing fewer than 30% of the eligible post-hospitalized cardiac population [22], [23].

### Data Sources

The Socio-Economic Status and Acute Myocardial Infarction Study (SESAMI) study is a prospective, observational investigation of patients hospitalized for AMI between December 1, 1999 and February 28, 2003 in 53 large volume acute hospitals throughout Ontario, Canada. [15] Details about SESAMI have been previously published. [2], [15], [24] Briefly, the study consisted of baseline surveys, in-hospital chart abstraction, and telephone follow-up at 30-days and one-year post AMI. Mortality over the 9.6 year follow-up was assessed using vital statistics data (the Registered Persons Data Base), as has been used previously and whose accuracy has been verified [2], [13], [15], [25].

### Study Sample

Details of SESAMI recruitment and eligibility have been previously described. [15] All patients were English-speaking and were enrolled if 2 of 3 AMI criteria were met: presence of symptoms, abnormal electrocardiographic findings (ST elevation or depression), or elevated serum levels of cardiac enzymes (CK-MB and/or Tropinin I levels). Patients were excluded if they were <19 or >101 years of age, lacked a valid health card number issued by the province of Ontario, or were transferred to the recruiting hospital. In total, 2829 consecutive participants were enrolled and underwent detailed clinical information abstracted from medical charts pertaining to the index hospitalization. Given severe access constraints and significant waiting-time delays for multidisciplinary secondary prevention programs, this sub-study required that all SESAMI patients survive for at least one year following AMI to ensure each patient had equal opportunity for referral and participation into the program. All patients had to be available and agree to participate in follow-up interviews at one-year to evaluate self-reported functional capacity, medication compliance, psychosocial status, and quality of life (see below). Among the 1859 (65.7%) remaining patients who were alive and eligible for the one-year follow-up telephone interview, 1463 (78.7%) patients participated; 95 patients were excluded because of missing data, leaving 1368 patients available for final analyses. Despite attrition due to death and follow-up, previous work has determined that the distribution and prevalence of ethno-demographic and comorbid characteristics across income and education categories were similar between the current study sample and the original SESAMI cohort from which it was derived. [26] The Sunnybrook Health Sciences Centre Research Ethics Board approved the study protocol and methodology and all subjects gave informed consent to participate.

### Socioeconomic Status

Previous studies have demonstrated the importance of self-reported income as an independent determinant of mortality after AMI. Accordingly, annual self-reported income served as our primary socioeconomic indicator for this study. Self-reported household annual income (from all sources) in Canadian (C) dollars was ascertained using a 7-level categorical scale ranging from <C$15 000 to >C$80 000; income categories were then re-aggregated into three age-specific categories (i.e., <$30 000; $30000-$59999; $60000+ for patients younger than 65 years; <$20000; $20000-39 999; $40000+ for patients 65 years and older), as has been done previously. [2] These cut-points corresponded to the low, medium, and high-income taxation thresholds for Canadian citizens in the labour force, as previously described. [15] A repeat analysis in which income aggregation ignored age-specific income rankings did not alter our results.

Our study also collected information on education. Self-reported educational status incorporated a 5-level categorical variable ranging from incomplete high school to university degree. All our analyses examining income-mortality associations adjusted for patient-level education. However, as a sensitivity analysis, we re-analyzed our data using education (as opposed to income) as our primary SES indicator. While the magnitude of association between unadjusted education and mortality was smaller than that for income, the relationships between education, functional recovery, and post-AMI survival were similar as for income.

### Other Baseline Characteristics

Information on ethnicity was obtained via self-report from one or more categories of 13 ethno-racial subgroups. [27] For the purposes of this study, ethno-racial data were re-aggregated *a priori* into five variables: White, Black, South Asian, First Nations, and Other (Other here includes East Asian/Chinese respondents), as in our previous studies. [26], [28] Several clinical and comorbid factors were identified and incorporated into the data base. We examined other clinical markers of disease severity (e.g., acute pulmonary edema, resting blood pressure, sinus tachycardia), cardiovascular risk factors (diabetes, hypertension, hyperlipidemia, and current or former smoking use), comorbidity (total number as well as type), [13], [29] during the index AMI hospitalization. In addition to these factors, we calculated the Global Registry of Acute Coronary Events (GRACE) prognostic index on each patient. The GRACE prognostic index was used to calculate a 6-month predicted post-AMI mortality risk-score based on age, development (or history) of heart failure, peripheral vascular disease, systolic blood pressure, Killip class, baseline serum creatinine concentration, elevated initial cardiac markers, cardiac arrest on admission, and ST segment deviation. The GRACE index has been previously validated in SESAMI patients. [28] Substituting the GRACE index with their original comprised clinical variables did not meaningfully alter the results.

### Multidisciplinary Secondary Prevention Service Delivery

Referrals to cardiac rehabilitation within the first year following hospital discharge were identified using self-report. All revascularization procedures (angioplasty or coronary bypass surgery), as well as physician visits (stratified according to physician specialty of general practitioner, internal medicine, and cardiology) were also assessed within the first year following the index AMI hospitalization. [30] We examined the prescribing of cardiovascular medications (aspirin, beta-blockers, statins, ACE inhibitors, and nitrates) at hospital discharge. We also assessed the utilization of, and adherence to, cardiovascular medications throughout the year following hospitalization on the assumption that self-management behaviours reflect the quality and effectiveness of secondary prevention service delivery. The utilization of, and adherence to, pharmacological therapies over the first year were ascertained through serial telephone interviews in which patients were asked to collect and read the names of all medications currently taken. There was moderate to good agreement between self-reported medication use and drug-claims for SESAMI patients aged 65 years and older for which drug claims data were available (Kappas ranging from 0.43 to 0.60 for beta-blockers and statins, respectively).

### Functional Recovery

Functional recovery was assessed using the Duke Activity Status Index (DASI), as measured at baseline (i.e., 30 days post-AMI) and at follow-up (i.e., 1-year post AMI), and expressed as peak oxygen consumption (peak VO_2_). [31] The DASI questionnaire and its derived functional capacity, expressed as ml/kg/min, have been validated against objectively measured peak VO_2_ from cardiopulmonary exercise testing, [32], [33] and therefore, served as our primary indicator for functional recovery. (See Appendix S1).

As other surrogates of functional recovery, we examined changes in psychosocial stress, including depression, social support, chronic stress, as well as other measures of self-rated physical and mental health status at 30-days and one-year after AMI. Chronic stress incorporated the National Population Health Survey questions related to stressful life events. [34] Self-rated physical and mental health status was assessed using the short-form 12 questionnaire while depression was assessed using the Brief Carroll Depression Rating Scale [35]–[37].

### Outcome

Long-term mortality (as of December 31, 2010, representing a mean follow-up of 9.6 years) served as the primary outcome for our study, which corresponded to 11,765 patient life-years of follow-up. No patients were lost to follow-up.

### Statistical Analysis

Income was analyzed as a continuous variable, to examine the main-effect of income across the 3 income tertiles using one degree of freedom, and categorically to allow for the comparison between tertiles, where overall income associations where statistically significant. The Mantel-Haenszel test for trend was used for categorical data and ANOVA (or nonparametric tests where relevant) were used for continuous data to detect differences in baseline characteristics between income categories. Multiple Least Squares Regression analyses (using backward stepwise regression) were used to examine the relationship between SES and self-reported functional recovery, after adjusting for all baseline characteristics (including age, sex, baseline functional capacity, cardiac risk, comorbidity, chronic stress, depression, and medication use) as well as for referrals and use of cardiac specialty services (including cardiac rehabilitation referral, cardiology visits, cardiac procedures, and evidence-based medications).

Cox proportional hazards models were used to examine which factors throughout the first year of AMI recovery were most strongly associated with long-term survival irrespective of patient SES, cardiac specialty use, or cardiac rehabilitation referrals. The mortality hazard associated with each dataset variable including SES, ethnicity, rurality, age, sex, cardiac risk factors, prior medical history, total numbers and types of medical comorbidities, predicted 6 month mortality (using the GRACE predictive risk index), medications at hospital discharge, as well as primary care and specialty care physician visits, coronary interventions, medication adherence, changes in quality of life, depression, chronic stress, and changes in functional capacity during the year of AMI follow-up were assessed using backwards stepwise regression.

To examine the extent to which baseline and follow-up factors modulated or altered the relationship between SES and mortality, sequential risk adjustment was undertaken for each baseline and follow-up factor using backward stepwise regression techniques, while forcing income into each mortality model. To quantify the relative contribution of functional recovery to the observed association between income and mortality, we used the formulae:
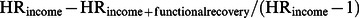
(38)


The relative contribution of functional recovery on income-mortality associations were examined incrementally over and beyond other baseline and recovery factors (i.e,. all models adjusted for self-reported functional capacity, self-reported physical health, emotional health, chronic stress, depression at baseline, as well as one-year changes in chronic stress and depression. However, given the high correlation between the DASI and SF-12 self-rated physical health measures (r = 0.73, P<0.001), a risk-adjustment model did not include change scores for both the DASI and the SF-12 self-rated physical health score within the same statistical model. Statistical models in which functional recovery were derived from changes in DASI yielded similar results as those in which functional recovery were derived using the SF-12 self-rated physical health composite score. Formal diagnostic testing revealed no evidence of multi-collinearity in any of our statistical models. A sensitivity analysis using non-parsimonious modeling did not meaningfully alter our results. We tested for violations of the proportionality assumption in all proportional hazard model specifications. All analyses were performed using SAS statistical software, version 9.1 (SAS Institute, Cary, NC).

## Results

### Baseline Characteristics

Socioeconomically disadvantaged patients were significantly older, more likely to be women, have fewer social supports, greater comorbidities, and higher predictive 6-month mortality rates than their more affluent counterparts. Income disadvantaged patients were also significantly less likely to receive beta blockers and more likely to receive nitrates at hospital discharge **(** **Table 1** **)**.

**Table 1:** Baseline characteristics of study participants according to income tertile.

	Income			
	Low(N = 331)	Intermediate (N = 472)	High(N = 565)	P value
**ETHNO-GEO-DEMOGRAPHIC**				
Age in years, mean (STD)	65.1 (12.4)	63.9 (12.4)	60.5 (12.1)	<0.001
Sex, female (%)	157 (47.4)	127 (26.9)	112 (19.8)	<0.001
Caucasian (%)	252 (76.1)	405 (85.8)	517 (91.5)	<0.001
Rural residence (%)	28 (8.5)	28 (5.9)	18 (3.2)	<0.001
**PSYCHOSOCIAL**				
Lives alone (%)	100 (30.5)	86 (18.3)	46 (8.2)	<0.001
Chronic stress, mean (STD)	2.8 (2.7)	2.4 (2.1)	2.2 (2.0)	0.05
Education (%)				
Incomplete high-school	171 (52.5)	162 (34.5)	96 (17.0)	<0.001
Complete high-school	76 (23.3)	158 (33.6)	181 (32.1)	
University or college degree	79 (24.2)	150 (31.9)	287 (50.9)	
**CLINICAL CHARACTERISTICS**				
Predicted 6 month mortality rate, mean (STD)	3.45 (4.3)	3.13 (3.96)	2.51 (3.8)	0.01
Heart rate on admission, mean (STD)	82.2 (23.0)	80.6 (23.2)	79.2 (22.3)	0.04
Systolic blood pressure on admission, mean (STD)	150.7 (30.9)	148.2 (31.9)	147.2 (30.2)	0.01
Diastolic blood pressure on admission, mean (STD)	83.3 (18.5)	83.4 (19.1)	84.8 (19.1)	0.77
Respiratory rate on admission, mean (STD)	20.3 (4.8)	19.7 (4.3)	19.3 (4.4)	0.01
Acute pulmonary edema on admission (%)	9 (2.7)	12 (2.5)	10 (1.8)	0.32
ST elevation myocardial infarction (%)	128 (39.0)	193 (40.9)	252 (44.8)	0.08
Total number of comorbid conditions, mean (STD)	2.23 (0.93)	2.1 (0.99)	1.95 (1.03)	0.03
Previous AMI (%)	93 (28.1)	121 (25.6)	113 (20.0)	0.004
Previous angina (%)	167 (20.2)	223 (47.3)	241 (42.7)	0.02
Previous Heart failure (%)	67 (20.2)	67 (14.2)	61 (10.8)	<0.001
Diabetes (%)	100 (30.2)	107 (22.7)	96 (17.0)	<0.001
Hypertension (%)	177 (53.5)	213 (45.1)	248 (43.9)	0.009
Hyperlipidemia (%)	124 (37.5)	193 (40.9)	246 (43.6)	0.07
Smoking (%)	132 (39.9)	183 (38.8)	209 (37.0)	0.37
Asthma (%)	31 (9.4)	27 (5.7)	22 (3.9)	0.001
COPD (%)	67 (20.2)	44 (9.3)	40 (7.1)	<0.001
Cancer (%)	2 (0.6)	7 (1.5)	11 (1.95)	0.11
Dementia (%)	6 (1.8)	3 (0.6)	1 (0.18)	0.007
Dialysis (%)	1 (0.3)	2 (0.42)	1 (0.18)	0.67
Peripheral artery disease (%)	21 (6.3)	31 (6.6)	31 (5.5)	0.55
Stroke or TIA (%)	10 (3.0)	20 (4.2)	22 (3.9)	0.58
Previous depression (%)	26 (7.0)	16 (3.4)	19 (3.4)	0.004
**PROCESSES OF CARE DURING HOSPITALIZATION**				
Length of stay during index hospitalization, mean (STD)	8.9 (5.6)	8.9 (6.5)	8.5 (5.3)	0.67
Aspirin on discharge (%)	233 (70.4)	352 (74.6)	426 (75.4)	0.12
Nitrates on discharge (%)	126 (38.1)	139 (29.5)	169 (29.9)	0.02
Beta blockers on discharge (%)	218 (65.9)	337 (71.4)	414 (73.3)	0.02
Statins on discharge (%)	181 (54.7)	258 (54.7)	316 (55.9)	0.69

### SES and Functional Recovery

Socially disadvantaged patients had poorer baseline self-reported functional capacity and achieved less improvement in one-year post-AMI functional recovery than did their higher SES counterparts. Patients of higher incomes also experienced better recovery from chronic stress, depression, self-rated physical and mental health than did patients who had lower annual earnings (P<0.001 for all), although the magnitude of changes for all of these other variables were less marked than the DASI-derived self-reported functional capacity. **(** **Table 2** **)**.

**Table 2:** Functional recovery, depression, psychosocial stress, emotional and physical well-being according to income tertile during the year following AMI hospitalization.

	Low income(N = 331)	Intermediate income (N = 472)	High income(N = 565)	P value
**Functional recovery (Duke Activity Status Index)**				
Baseline V02 peak in ml/kg/min, mean score (STD)	15.4 (4.2)	17.4 (4.8)	18.6 (5.1)	<0.001
Change in VO2 peak in ml/kg/min between 30-days and 1-year after AMI, mean score (STD)	2.1 (5.1)	3.2 (5.8)	4.5 (5.7)	<0.001
**Depression (Carroll-Depression Inventory)**				
Baseline Chronic Depression Inventory, mean score (STD)	0.19 (0.4)	0.12 (0.33)	0.08 (0.27)	0.01
One-year changes in Chronic Depression Inventory following hospitalization, mean score (STD)	−0.76 (0.44)	−0.08 (0.34)	−0.02(0.28)	0.03
**Chronic stress**				
Baseline chronic stress, mean score (STD)	2.8 (2.7)	2.4 (2.1)	2.2 (2.0)	<0.001
One-year change in chronic stress following hospitalization, mean score (STD)	−0.14 (2.3)	−0.025 (2.1)	0.08 (1.0)	0.12
**Emotional well-being (SF-12)**				
Baseline SF-12 emotional, mean (STD)	17.1 (3.9)	18.1 (3.6)	18.5 (3.4)	0.03
One-year change in SF-12 emotional following hospitalization, mean score (STD)	0.61 (3.9)	0.99 (3.4)	1.2 (3.4)	0.01
**Physical well-being (SF-12)**				
Baseline SF-12 physical, mean score (STD)	12.9 (3.3)	13.8 (3.2)	14.4 (3.1)	<0.001
Changes in SF-12 physical, following hospitalization, mean score (STD)	0.98 (3.5)	1.5 (3.3)	2.0 (3.2)	<0.002

**Figure 1:**
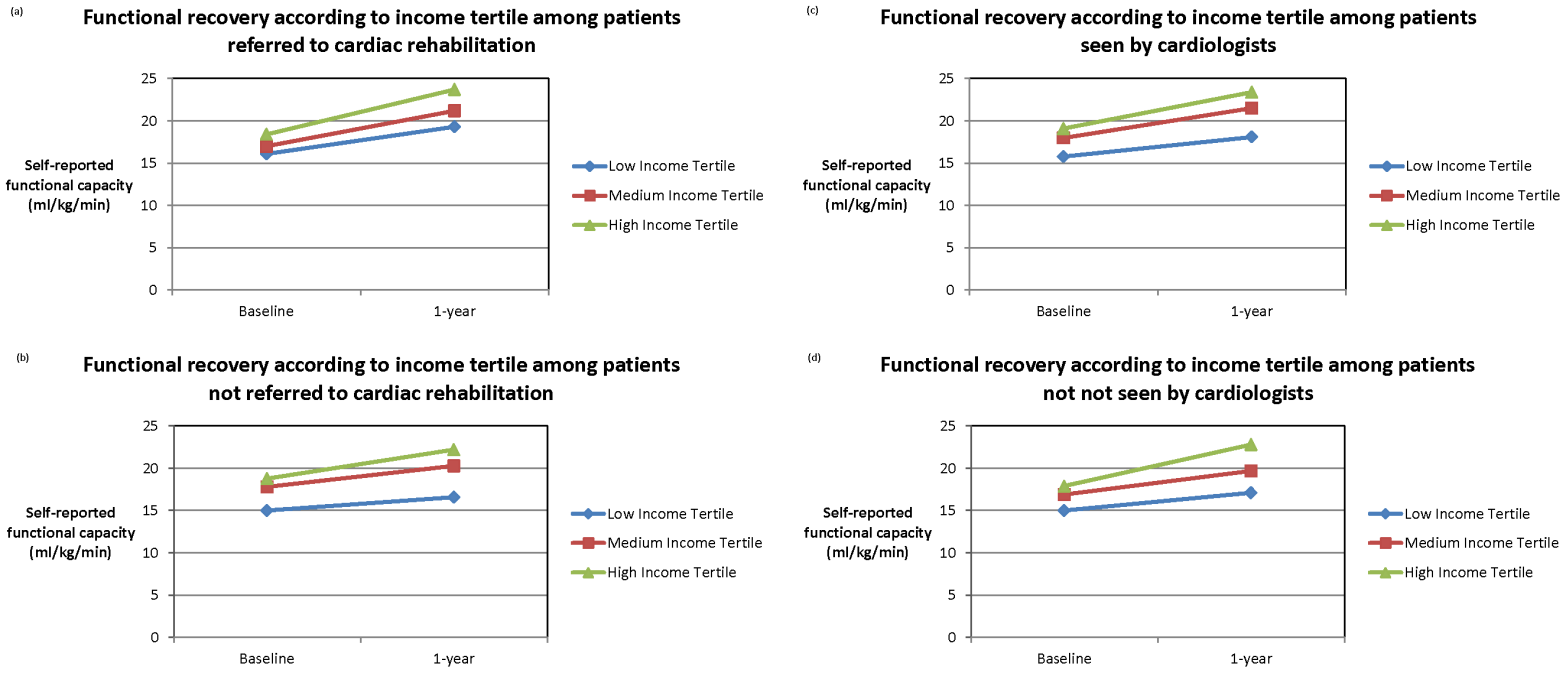
Function recovery according to income tertile among patients referred to cardiac rehabilitation (Figure 1a), not referred to cardiac rehabilitation (Figure 1b), seen by a cardiologist in follow-up (Figure 1), not seen by a cardiologist in follow-up (Figure 1d).

Functional recovery improved among all patients regardless of SES or referral to cardiac rehabilitation, but did so more markedly among patients in higher SES tertiles (i.e. highest SES tertile patients on average, experienced a 1.80 ml/kg/min increase in peak V02 as compared with lowest SES tertile patients, P<0.001) **(** **Figure 1** **)**, and did so even after adjustment for all baseline factors irrespective of whether functional recovery was assessed as a continuous or a categorical variable. For example, patients in lowest as compared with highest income tertile patients were 44% less likely to experience functional recovery gains exceeding levels corresponding to the sample median, even after adjusting for all remaining factors (Adjusted OR: 0.56; 95% CI:0.38–0.84, P = 0.005).

### SES and Secondary Prevention Services

Patients within highest income tertiles were 60% more likely to be referred to cardiac rehabilitation than those in lowest income tertiles. Income disadvantaged patients were significantly less likely to be followed up by a cardiologist, to receive cardiac rehabilitation, and to be taking evidence-based pharmacotherapies (B-blockers, aspirin, statins, and ACE inhibitors) during the year following AMI than were their higher SES counterparts**. (** **Table 3** **)**.

**Table 3:** Health service delivery according to income tertile during the year following AMI hospitalization.

	Low income(N = 331)	Intermediate income (N = 472)	High income(N = 565)	P value
**Cardiac rehabilitation**				
Cardiac rehabilitation participation by 30-days post-hospitalization (%)	93 (29.3)	151 (32.4)	242 (43.7)	<0.001
Cardiac rehabilitation participation by 1-year post-hospitalization (%)	120 (37.3)	213 (45.7)	333 (59.6)	<0.001
**Cardiology visits**				
Cardiology visit within 30-days of hospitalization (%)	163 (50.3)	243 (51.8)	312 (55.5)	0.11
Cardiology visit within 1 year of hospitalization (%)	261 (78.9)	381 (81.4)	510 (90.3)	<0.001
**Internal Medicine visits**				
Internal medicine visit within 30-days of hospitalization (%)	24 (7.7)	39 (8.7)	59 (9.0)	0.55
Internal medicine visit within 1 year of hospitalization (%)	47 (17.2)	94 (19.5)	94 (16.1)	0.66
**General Practice visits**				
GP visit within 30-days of hospitalization (%)	267 (81.4)	397 (84.1)	466 (82.5)	0.80
GP visit within 1 year of hospitalization (%)	323 (97.6)	456 (96.6)	551 (97.5)	0.92
**Cardiac interventions**				
Percutaneous Coronary Intervention within 30 days of hospitalization (%)	81 (25.6)	116 (25.0)	196 (34.8)	0.001
Percutaneous Coronary Intervention within 1 year of hospitalization (%)	99 (29.9)	151 (32.0)	223 (39.5)	0.002
Coronary artery bypass surgery within 30 days of hospitalization (%)	43 (13.4)	65 (13.9)	78 (13.9)	0.85
Coronary artery bypass surgery within 1 year of hospitalization (%)	59 (17.8)	88 (18.6)	113 (20)	0.41
**Beta Blockers**				
No B-blockers taken at 30-days or at 1 year (%)	44 (13.3)	40 (8.5)	29 (5.1)	<0.001
B-blockers taken at 30-days but not at 1 year (%)	29 (8.8)	45 (9.5)	48 (8.5)	
B-blockers taken at 1 year but not at 30-days (%)	26 (7.9)	35 (7.4)	34 (6.0)	
B-blockers taken at 30-days and 1 year (%)	232 (70.1)	352 (74.6)	454 (80.4)	
**ACE Inhibitors**				
No ACE inhibitors taken at 30-days or at 1 year (%)	73 (22.1)	76 (16.1)	92 (16.3)	0.01
ACE inhibitors taken at 30-days but not at 1 year (%)	30 (9.1)	46 (9.8)	41 (7.3)	
ACE inhibitors taken at 1 year but not at 30-days (%)	43 (13.0)	93 (19.7)	79 (14.0)	
ACE inhibitors taken at 30-days and 1 year (%)	185 (55.9)	257 (54.5)	353 (62.5)	
**Statins**				
No statins taken at 30-days or at 1 year (%)	67 (20.2)	94 (19.9)	96 (17.0)	0.008
Statins taken at 30-days but not at 1 year (%)	37 (11.2)	43 (9.1)	42 (7.4)	
Statins taken at 1 year but not at 30-days (%)	52 (15.7)	75 (15.9)	71 (12.6)	
Statins taken at 30-days and 1 year (%)	173 (52.9)	260 (55.1)	356 (63.1)	
**Aspirin**				
No Aspirin taken at 30-days or at 1 year (%)	29 (8.8)	26 (5.5)	27 (4.8)	0.01
Aspirin taken at 30-days but not at 1 year (%)	22 (6.7)	37 (7.8)	35 (6.2)	
Aspirin taken at 1 year but not at 30-days (%)	35 (10.6)	46 (9.8)	46 (8.1)	
Aspirin taken at 30-days and 1 year (%)	245 (74.0)	363 (76.9)	457 (80.9)	
**Nitrates**				
No Nitrate taken at 30-days or at 1 year (%)	160 (58.3)	247 (52.3)	332 (58.8)	<0.001
Nitrates taken at 30-days but not at 1 year (%)	67 (20.2)	114 (24.2)	128 (22	
Nitrates taken at 1 year but not at 30-days (%)	35 (10.6)	40 (8.5)	52 (9.2)	
Nitrates taken at 30-days and 1 year (%)	69 (20.9)	71 (15.0)	53 (9.4)	

### Secondary Prevention Services and Functional Recovery

Neither cardiac rehabilitation referrals nor specialty care visits were significantly associated with functional recovery after adjusting for all baseline factors. Among all secondary prevention factors examined, only 30-day post-AMI coronary revascularization (PCI or CABG) significantly predicted functional recovery after AMI (P<0.001).

### SES, Functional Recovery and Long-term Mortality

After adjusting for baseline and follow-up factors, functional recovery was the strongest modifiable predictor of long-term mortality based on the rank-order magnitude of the Chi-Square, and remained so irrespective of SES strata, cardiac rehabilitation referral or physician specialty service use (interaction terms between SES strata or cardiac rehabilitation referral or physician specialty service utilization, functional recovery, and mortality were all P>0.5). Each 1 ml/kg/min increase in estimated peak V0_2_ was associated with a 9% reduction in long-term mortality (Adjusted HR: 0.91; 95% CI: 0.88–0.94, P<0.001).

There was a strong association between income and long-term mortality (Unadjusted HR for income with one-degree of freedom: 0.62; 95% CI: 0.54–0.71, P<0.001) was attenuated by 42% after adjustment for all post-AMI baseline and follow-up variables, excluding functional recovery (Adjusted HR: 0.78; 95% CI:0.65–0.93; P = 0.005). Adding functional recovery further reduced the magnitude of this association explaining an additional 27% of income’s association with mortality, rendering the relationship between income and mortality no longer statistically significant (Adjusted HR: 0.84; 95% CI:0.70–1.00, P = 0.05) **(** **Table 4** **)**. In contrast, sequential risk-adjustments for access to cardiac rehabilitation and specialty service had no significant impact on SES-mortality associations.

**Table 4:** The relationship between income and long-term survival after sequential adjustments for factors associated with one-year recovery^i^.

Model	Income	Hazard Ratio +/−95%Confidence Interval)	95% ConfidenceInterval
Unadjusted model^ii^			
	Low income	2.19 (1.69–2.83)	<0.001
	Medium income	1.59 (1.24–2.04)	<0.001
	High income	1.00 (Reference)	Reference
	Overall wealth-mortality-gradientiii	0.62 (0.54–0.71)	<0.001
Adjusted for all baseline and follow-up factors with the exception of functional recovery^iv^			
	Low income	1.46 (1.06–2.03)	0.02
	Medium income	1.45 (1.07–1.96	0.02
	High income	1.00 (Reference)	Reference
	Overall wealth-mortality-gradient^‡^	0.78 (0.65–0.93)	0.005
Adjusted for all baseline and follow-up factors as well as functional recovery^v^			
	Low income	1.40 (1.00–1.95)	0.05
	Medium income	1.33 (0.99–1.81)	0.06
	High income	1.00 (Reference)	Reference
	Overall wealth-mortality-gradient^‡^	0.84 (0.70–1.00)	0.05

i Functional recovery was defined using self-reported DASI score. Statistical survival models incorporated Cox Proportional hazards and adjusted for clinical and process factors using backward stepwise regression.

ii The unadjusted mortality model examines the crude relationship between income and long-term mortality with no adjustment for any concomitant factors.

iii Overall wealth-mortality gradient examines income in tertiles but with one degree of freedom.

iv The partially adjusted mortality model examines the relationship between income and long-term mortality after adjustments for age, sex, education, ethnicity, rurality, predicted 6 month mortality from the time of hospitalization, hypertension, diabetes, hyperlipidemia, comorbidities, smoking history, social isolation, history of depression, depression at 30-days, depression change between 30-days and 1-year, quality of life (SF-12) at 30-days and changes between 30-days and 1-year, chronic stress at 30-days and changes between 30-days and 1-year, Percutaneous Coronary Intervention within 1 year of hospitalization, Coronary artery bypass surgery within 1 year of hospitalization, physician visits (cardiologist, internal medicine and general practitioner), cardiac rehabilitation referral. as well as pharmacotherapies (beta-blockers, statins, ACE inhibitors, aspirin, nitrates) at hospital discharge, 30-days, and 1 year post-MI.

v All factors included in the partially adjusted mortality model+functional capacity at 30-days and changes in functional capacity between 30-days and 1-year.

**Figure 2:**
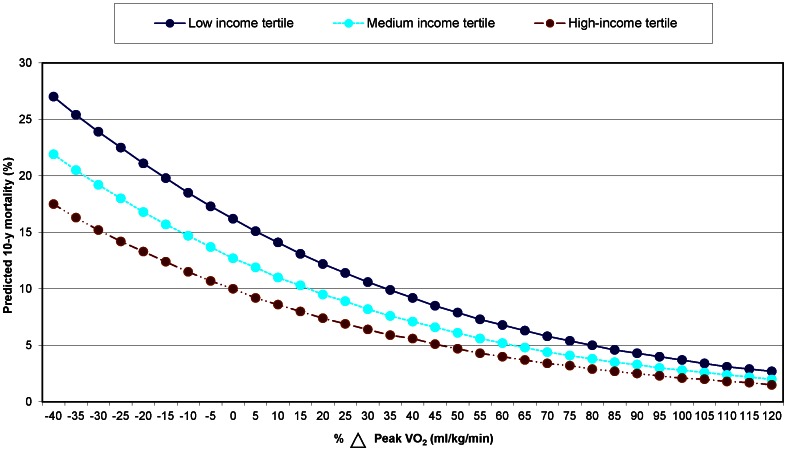
Relationship between functional recovery (i.e., % 1-year changes in self-reported peak VO_2)_ and expected 10-year mortality according to income after risk-adjustment for all remaining factors.

After adjusting for all factors, lowest income-tertile patients whose functional recovery exceeded that of the sample median had similar predicted long-term mortality as high-income tertile patients whose functional recovery improvements were less than the 20^th^ percentile. **(** **Figure 2** **)**.

## Discussion

Our study demonstrated that higher SES patients experienced significantly greater post-AMI functional recovery than did their socioeconomically disadvantaged counterparts. Functional recovery was the strongest modifiable predictor of long-term mortality irrespective of SES, and explained nearly 30% of the association between SES and long-term mortality after AMI, as demonstrated through sequential risk-adjustment. The effects of functional recovery on SES-mortality associations were not explained by access inequities to physician specialists or cardiac rehabilitation.

Our results are consistent with other studies which have demonstrated that patients of lower SES have poorer functional capacity. [39]–[41] For example, Shishehbor and colleagues in which differences in functional capacity explained as much as 47% of the SES-mortality associations among patients with suspected coronary artery disease. [14] Moreover, the 9% reduction in long-term mortality associated with each increased calculated MET, as derived using a self-reported functional capacity survey is comparable to studies that examined the relationship between METs and survival as measured objectively from exercise testing [42].

Our study builds upon previous studies by examining the relationship between SES and functional recovery during the transitional year of AMI convalescence, where the baseline risk of death and the needs for specialized cardiovascular services are highest. Our study also examined functional recovery within a context of other psychosocial, clinical, process of care and self-rated physical and mental health measures. The consistency by which SES correlated with functional recovery and the magnitude by which self-reported functional recovery explained SES-mortality associations underscores the importance of physical activity and exercise as social determinants of cardiovascular health.

We had hypothesized that SES access inequities to specialized cardiovascular services, such as cardiac rehabilitation and physician specialists, might have explained why socially-disadvantaged patients experience fewer gains in functional recovery after AMI as compared with their socially-advantaged counterparts. However, such was not the case. While patients in lowest income tertiles were 60% less likely to be referred to cardiac rehabilitation following AMI, cardiac rehabilitation was not independently associated with functional recovery after adjusting for patient factors. Indeed, functional recovery remained systematically lower among socially-disadvantaged irrespective of access to cardiac rehabilitation and/or cardiac specialists, which may partially explain why access to specialized cardiac services did not explain post-AMI SES-mortality associations.

Socioeconomically disadvantaged patients may experience poorer post-AMI functional recovery for several reasons. First, available evidence has shown that lower socioeconomic patients are generally less behaviourally engaged in healthy lifestyle choices, [43] in part, due to poorer awareness and insights into their health and disease. [44] Second, some have argued that socioeconomically-disadvantaged patients may have fewer social supports and networks. [45] Such networks may serve to act on the community culture of healthy life-style living, [7] resulting in such patients participating less frequently in physical activity and exercise as compared with their more affluent counterparts. [45] Third, socioeconomically-disadvantaged patients may be functionally limited by other co-existing medical illnesses and/or disabilities, which impede the ability of a patient to exercise. [46] Finally, lower SES patients may be challenged by employment constraints or finances to gain access to community resources and/or exercise accessories [47].

Our results support the need for innovative solutions to improve exercise and physical activity patterns among socio-economically disadvantaged patients. However, such innovative solutions may not necessarily simply reside with the broader implementation of established health services, such as cardiac rehabilitation programs and access to physician specialists. Instead, such strategies may necessitate other health and social policies, which may necessitate more integrative solutions into the workplace, tax-incentives, community-networks, and investments into the built-environment.

Our study has several important limitations which warrant discussion. First, functional recovery data were obtained using self-reported Questionnaires. While the functional capacity derived from DASI has been validated, [32], [33] and while our study’s use of the DASI questionnaire yielded similar results as did the self-rated physical health score as derived from the SF-12, it is possible that our findings may have differed had we estimated or directly measured peak VO_2_ during progressive exercise testing. Second, ours was an observational study and some clinical details, such as left ventricular function were unavailable. Moreover, all of our survey data was confined to the first year of AMI recovery. We acknowledge that residual unmeasured confounding, particularly throughout the multiple years of follow-up, might have partially explained our results. That being said, our study did adjust for over 40 clinical, psychosocial, and process of care. Furthermore, we believe that the magnitude of associations between factors collected during the year following the index AMI and survival throughout the many years that follow would have if anything attenuated over time. Therefore, we believe that the associations between SES, functional recovery, and long-term mortality are conservative. Moreover, available evidence has demonstrated that the transitional period following AMI is important given the prevalence of cardiovascular specialty care-gaps, fragmentation and discontinuity in health care delivery as patients navigate from hospitals to community-based ambulatory care settings. [15], [48]–[51] Finally, our study was conducted among a sample of AMI patients who survived and participated in one year interviews. While the distribution of sociodemographic factors among our AMI sub-sample was similar to the original SESAMI cohort, [26] the extent to which our results are applicable to all AMI populations remains unclear. That said, the original SESAMI cohort did enrol 70% of consecutive AMI patients from 95% of the large volume hospitals throughout Ontario - - a province which comprises 40% of the Canadian population. [26] These limitations must be counter-balanced against the strengths of this study, which include the comprehensiveness of our clinical, psychosocial, behavioural, and health service utilization data, as well as the duration and completeness of follow-up.

In conclusion, our study demonstrated the importance of functional recovery on explaining long-term SES-mortality associations. Post-AMI functional recovery may therefore represent an important intermediary causal pathway determinant of SES-outcome gradients after AMI. Given that the relationships between SES, functional recovery, and outcomes occurred independently of, and irrespective to, exposure to specialty cardiac services, innovative solutions must look beyond improvements in access to cardiac rehabilitation to improve SES-outcomes gradients after AMI. Such solutions may require novel policies that better integrate physical activity and exercise-based interventions into communities to better target and improve functional recovery and outcomes among socioeconomically-disadvantaged populations.

## Supporting Information

Appendix S1 The Duke Activity Status Index is a self-administered questionnaire that measures a patient's functional capacity. It can be used to estimate the patient’s peak oxygen uptake.
(DOC)
