# Supplementary material for: Socioeconomic Status, Functional Recovery, and Long-Term Mortality among Patients Surviving Acute Myocardial Infarction
Source: PLoS One. 2013 Jun 3;8(6):e65130. doi: 10.1371/journal.pone.0065130 (PMC3670842; doi:10.1371/journal.pone.0065130)
Supplement: Appendix S1 — The Duke Activity Status Index is a self-administered questionnaire that measures a patient's functional capacity. It can be used to estimate the patient’s peak oxygen uptake. (DOC) [file pone.0065130.s001.doc]

**Appendix S1** The Duke Activity Status Index is a self-administered questionnaire that measures a patient's functional capacity. It can be used to estimate the patient’s peak oxygen uptake.

| **Item** | **Activity** | **Yes** | **No** |
| --- | --- | --- | --- |
| 1 | Can you take care of yourself (eating dressing bathing or using the toilet)? | 2.75 | 0 |
| 2 | Can you walk indoors such as around your house? | 1.75 | 0 |
| 3 | Can you walk a block or two on level ground? | 2.75 | 0 |
| 4 | Can you climb a flight of stairs or walk up a hill? | 5.50 | 0 |
| 5 | Can you run a short distance? | 8.00 | 0 |
| 6 | Can you do light work around the house like dusting or washing dishes? | 2.70 | 0 |
| 7 | Can you do moderate work around the house like vacuuming sweeping floors or carrying in groceries? | 3.50 | 0 |
| 8 | Can you do heavy work around the house like scrubbing floors or lifting and moving heavy furniture? | 8.00 | 0 |
| 9 | Can you do yardwork like raking leaves weeding or pushing a power mower? | 4.50 | 0 |
| 10 | Can you have sexual relations? | 5.25 | 0 |
| 11 | Can you participate in moderate recreational activities like golf bowling dancing doubles tennis or throwing a baseball or football? | 6.00 | 0 |
| 12 | Can you participate in strenuous sports like swimming singles tennis football basketball or skiing? | 7.50 | 0 |

Duke activity status index = SUM(values for all 12 questions)

Interpretation: • maximum value 58.2 • minimum value 0

Estimated peak oxygen uptake in mL/min = (0.43 * (duke activity status index)) + 9.6

References:

Hltaky MA Boineau RE et al. A brief self-administered questionnaire to determine functional capacity (The Duke Activity Status Index). Am J Cardio. 1989; 64: 651-654
